# COVID-19 Vaccine-Induced Thrombotic Thrombocytopaenia With Venous and Arterial Thrombosis: A Case Report

**DOI:** 10.7759/cureus.28535

**Published:** 2022-08-29

**Authors:** Zahid Khan, George Besis, Luciano Candilio

**Affiliations:** 1 Acute Medicine, Mid and South Essex NHS Foundation Trust, Southend on Sea, GBR; 2 Cardiology and General Medicine, Barking, Havering and Redbridge University Hospitals NHS Trust, London, GBR; 3 Cardiology, Royal Free Hospital, London, GBR

**Keywords:** covid-19-associated acute coronary syndrome, covid-19-induced thrombosis, ventricular dysrhythmia, timi flow, astra zeneca covid-19 vaccine, acute myocardial infarc, vaccine-induced thrombosis and thrombocytopenia (vitt)

## Abstract

Coronavirus disease 19 pandemic has put tremendous pressure on health systems and has caused significant morbidity and mortality throughout the world. Vaccination program against COVID-19 has been effective despite repeated outbreaks across the globe. It was however reported that COVID-19 vaccines in particular, the Oxford-AstraZeneca COVID-19 vaccine (AZD1222) was temporarily suspended by some European countries due to risk of thrombosis. COVID-19 is a prothrombotic condition and is associated with venous thromboembolism mainly. The condition can be challenging to diagnose due to its diagnostic variation. Cases of vaccine-induced thrombotic thrombocytopaenia has been reported in several countries. COVID-19 can also cause vaccine-induced thrombosis without thrombocytopaenia. The thrombotic events can affect different parts of the body including brain, heart, and peripheral vessels. We present a case of 54-year-old patient who presented with chest and abdominal pain for 12 hours and evidence of infero-lateral ST segment elevation on electrocardiogram. Patient received COVID-19 AstraZeneca vaccine 10 days prior to admission. Coronary angiography (CAG) showed occlusion of the proximal to mid part of the right coronary artery (RCA) distal to a large Right Ventricular branch with high thrombotic burden and multiple attempts at aspiration of the thrombus resulted in partial restoration of the flow to right coronary artery.

## Introduction

COVID-19 infection has caused significant mortality and morbidity since the beginning of the pandemic and vaccines have proved effective to minimize the damage. Various type of COVID-19 vaccines have been developed and as of 4th August 2022, 12.39 billion vaccines doses have been administered globally and up to 67.2% of the world population has received at least one dose of a COVID-19 vaccine [1]. Approximately, 6.94 million are now administered each day however it is important to mention that only 20.2% of people in low-income countries have received at least one dose in comparison to developed world [1]. Vaccine-related thromboembolic (VTE) complications have been reported with AstraZeneca Janssen) vaccines, mostly from Europe and North America [2,3].

The two common vaccines associated with a rare prothrombotic condition initially termed as thrombosis with thrombocytopaenia syndrome (TTS) and later as vaccine-induced immune thrombotic thrombocytopenia (VITT) were the Oxford-AstraZeneca and the Johnson & Johnson vaccines [4,5]. Due to the variation in its presentation, VITT can present a diagnostic uncertainty or challenge to healthcare professionals. The most common presentation ranges from constitutional symptoms to visual defects, severe headaches, leg and back pains, easy bruising, petechiae, myalgia, cerebral venous thrombosis and thrombosis at other sites [6,7].

The incidence of cerebral venous sinus thrombosis and other thrombosis along with thrombocytopaenia due to AstraZeneca and Johnson & Johnson vaccines occur most commonly within the first 4-28 days and many countries restricted their use in younger patients as a result [7]. Most evidence comes from case reports and case series although there are few systemic reviews and single centre studies also reported [5,8]. COVID-19 vaccines are also associated with other cardiac complications such as pericarditis and myocarditis [9]. Other side effects reported due to COVID 19 vaccination include anaphylaxis, appendicitis, Bell’s palsy, deep vein thrombosis, disseminated intravascular coagulation, encephalomyelitis, Guillain-Barre syndrome, haemorrhagic and non-haemorrhagic stroke, immune thrombocytopenia, narcolepsy, and transverse myelitis in general population [10]. Pfizer vaccine was associated with increased incidence of myocarditis/pericarditis and myopericarditis in patients whereas Moderna and AstraZeneca were associated with increased incidence of coronary thrombosis although Smadja et al. reported increased incidence of coronary thrombosis with Pfizer vaccine compared to Moderna and AstraZeneca vaccines [10,11].

We present a case of 54-year-old patient who presented with abdominal and chest pain for 12 hours. Electrocardiogram (ECG) showed ST elevation in inferior leads and coronary angiogram showed thrombosis in the right coronary artery (RCA). Patient had AstraZeneca vaccine 10 days prior to this episode and despite multiple aspiration attempts, only partial flow was achieved in the RCA and its side branches.

## Case presentation

**Figure 1:**
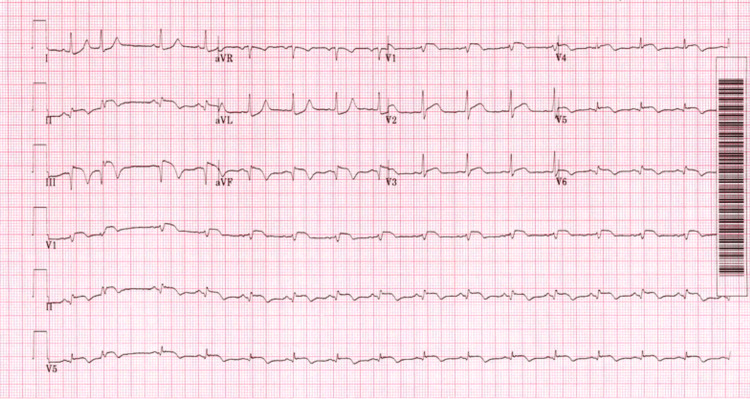
Electrocardiogram showing infero-lateral ST elevation

A 54-year-old-patient admitted with chest and abdominal pain for 12 hours and evidence of infero-lateral ST segment elevation on ECG to our cardiac centre for consideration of percutaneous coronary intervention (PCI) (Figure 1).

**Figure 2:**
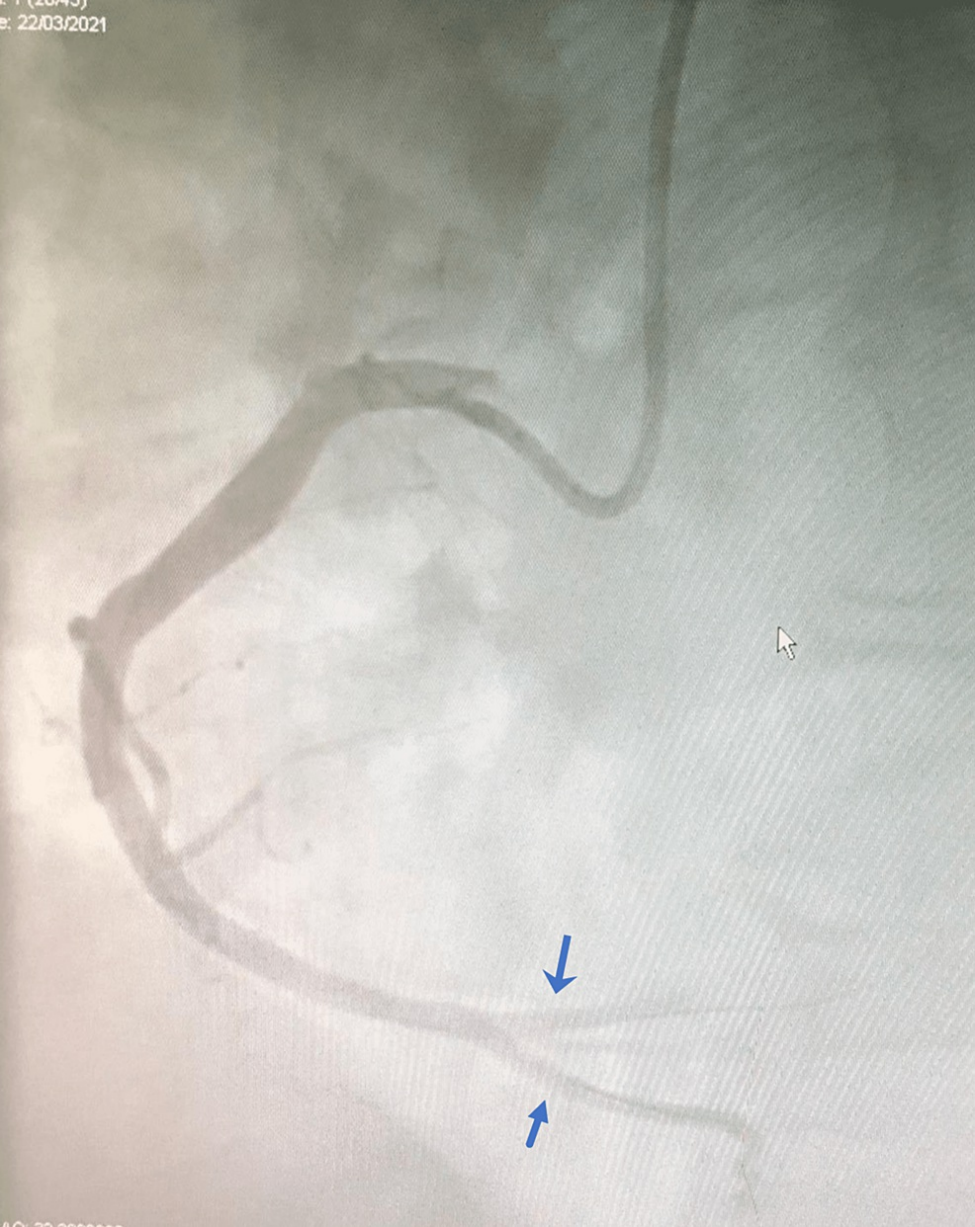
Coronary angiogram shows partial restoration of blood flow in RCA (TIMI 3 in posterior descending artery (PDA) and TIMI 1-2 in the posterior left ventricular (PLV) branch RCA: right coronary artery; TIMI: thrombolysis in myocardial infarction

Patient had no relevant past medical history and received COVID-19 vaccine AstraZeneca 10 days prior to admission. Coronary angiography (CAG) showed occlusion of the proximal to mid part of the RCA distal to a large right ventricular (RV) branch with high thrombotic burden. Multiple attempts at thrombus aspiration and ballooning resulted in partial flow restoration in RCA with thrombolysis in myocardial infarction (TIMI) 3 in posterior descending artery (PDA) and TIMI 1-2 in the posterior left ventricular (PLV) branch (Videos 1-2, Figure 2).

**Video 1:** Coronary angiogram showing right coronary artery thrombotic occlusion

**Video 2:** Coronary angiogram of the right coronary artery shows partial restoration of blood flow following multiple aspiration and ballooning attempts

Glycoprotein IIb-IIIa inhibitors (GPI) was given for 24 hours with a view to repeat CAG after 48 hours. Subsequent platelet count was noted to be 57x10^9^/l and GPI infusion was discontinued. Patient’s D-dimer was elevated at 56,693 ng/ml and peak troponin I 7263 ng/l and peak pro B-type natriuretic peptide (BNP) was 2027 pg/ml. Laboratory results for patient are shown in Table 1.

**Table 1:** Laboratory results trend for patient

Test	Day 1	Day 2	Day 4	Reference value
White cell count	22.41	16.98	22.87	3.5-11 x 10^9/l
Neutrophil	15.54	17.05	18.44	1.7-7.5 x 10^9/l
Haemoglobin	148	115	110	135-170 g/l
Platelet	57	38	22	140-400 x 10^9/l
Urea	8.7	9.6	12.7	2.9-8.2 mmol/l
Creatinine	126	243	106	66-112 µmol/l
Sodium	136	135	137	135-145 mmol/l
Potassium	4.0	6.2	4.2	3.5-5.1 mmol/l
C-reactive protein	67	79	156	0-5 mg/l
Troponin	2270	2758	7263	<14 ng/l
D-dimer	51,092	61,546	80,000	0-400 ng/ml
Alanine transaminase	156	161	141	7 to 56 U/l
Aspartate transferase	497	492	394	8 to 33 U/l
Bilirubin level	20	19	22	< 21 µmol/l
International normalized ratio (INR)	1.0	1.2	1.3	0.9-1.12
N-terminal pro-brain natriuretic peptide (pro-BNP)	1262	1840	2027	<400 ng/l

Patient unfortunately suffered a ventricular fibrillation (VF) cardiac arrest and return of spontaneous circulation (ROSC) was achieved with cardiopulmonary resuscitation and a single direct current (DC) shock. Platelet count dropped further to 42 x10^9^/l and 18 x10^9^/l on day 7 but fibrinogen level was normal. Computerized tomography head (CT) excluded intracranial bleed; however, patient had increased oxygen requirement the following day. Computerized tomography pulmonary angiography (CTPA) and computerized tomography abdomen and pelvis (CTAP) excluded pulmonary embolism but confirmed extensive thrombus in the superior mesenteric vein (SMV), main portal vein, left portal vein and splenic vein, with extensive circumferential oedema in the distal ileal loops. A disintegrin-like and metalloprotease with thrombospondin type 1 repeats (ADAMTS13 activity) level was 56.6 (Normal reference: 740-1420 ng/ml), anti-platelet factor 4 (anti-PF4) level was 0.760 (Normal reference: <0.50) and CD59 deficiency CD59, also known membrane inhibitor of reactive lysis (MIRL) level was 100. Autoimmune screening and protein electrophoresis was within normal range.

Urgent haematology consult was requested and provisional diagnosis of COVID vaccine-induced thrombocytopenia and arterial and venous thrombosis (VITT) was made based on positive platelet factor 4 antibodies level of 0.760. Patient was initially treated with intravenous hydrocortisone for thrombocytopaenia. Patient was intubated in view of rising oxygen requirement and type 1 respiratory failure, however unfortunately, patient suffered another asystolic cardiac arrest during induction of anaesthesia, patient sadly deceased despite best medical efforts.

## Discussion

Following the severe acute respiratory syndrome coronavirus (SARS-CoV-2) outbreak in China toward end of 2019, it was declared as a pandemic by World Health Organization (WHO) in 2020. Several vaccines were developed to prevent the outbreaks of the disease and to immunize population against it. The vaccines were reported to be safe and effective against the disease although concerns were raised against Oxford AstraZeneca vaccine [11]. COVID-19 was initially reported to be associated with pulmonary embolism only but subsequent studies confirmed it association with more widespread venous and arterial thromboembolism (ATE) [12,13]. The prevalence of VTE in COVID-19 patients admitted to intensive care unit (ITU) was reported between 0 and 69% and literature has shown significant lower burden of deep venous thrombosis (DVT) which leaves the peculiar question of pulmonary embolism unanswered [14].

Another study based on 89 patients admitted with coronary angiogram confirmed acute coronary syndrome (ACS), 37 patients had history of COVID-19 vaccination and the timing of vaccination dose to the actual event was <1, 1-2, 2-4, and >4 weeks in nine (24%), four (11%), 15(41%), and nine (24%) patients, respectively [5]. Twenty-eight (76%) patients had Oxford AstraZeneca vaccine and nine patients had Bharat Biotech’s Covaxin vaccine. There was no major difference in the baseline characteristics of the two groups except for symptoms to door time and thrombocytopaenia was noted in only two patients amongst the non-vaccinated group whereas none of the patients in the vaccinated group developed thrombocytopaenia. The thrombus burden was quite high in vaccinated group with reduced TIMI flow and in-patient and 30-day mortality was the same for both groups [5]. The incidence of venous thromboembolism (VTE and ATE were reported to be due to development of autoantibodies against platelet factor 4 antibodies in the vaccinated group in previous studies and the condition was described as VITT or TTS. This study showed that symptoms onset was usually within 5-10 days post-vaccination, and the cases were pretty much identical between 5 and 30 days post-vaccination [5].

VITT is associated with concurrent incidence of thrombosis and thrombocytopenia at the same time indicating that this is autoimmune process resulting in hypercoagulable state [15]. The incidence rate of VITT ranges from 1 in 125,000-1,000,000 individuals vaccinated for COVID-19 infection which makes it a very rare disease [16]. A systemic review showed that majority patients were young females aged 30-49 years, without any obvious comorbidities [6]. Females are at twice higher risk of HIT compared to males and this gender trend can be attributed to the clinical and pathophysiological resemblance of TTS with HIT [6]. This study also showed that males were more likely to develop arterial thrombi compared to female although venous thrombi were more common over arterial thrombi in general. It was also noted that risk factors associated with stasis and hypercoagulability components of the Virchow’s triad were more significant for venous thrombosis, whereas those associated with vessel wall damage or changes were more significant in arterial thrombosis [17].

A case report of an 86-year-old man with history of prostate cancer, paroxysmal atrial fibrillation (PAF), on apixaban 2.5 mg twice a day was admitted to hospital with a collapse, 30 minutes after receiving Pfizer-BioNTech vaccine [18]. ECGs showed inferior ST elevation and CAG revealed occlusion of the distal part of the left anterior descending coronary artery and distal part of the dominant RCA with large thrombus. PCI of the RCA with manual aspiration thrombectomy along with coronary balloon angioplasty was performed and patient was commenced on glycoprotein IIb/IIIa receptor inhibitor (eptifibatide) that improved the coronary arterial blood flow. This case most likely precipitated by thromboembolic event from Pfizer vaccine and patient unfortunately did not survive this event. Patient in our case report also had thromboembolic event 10 days after administration of AstraZeneca vaccine and partial flow was restored after several aspiration and ballooning attempts. The notable difference in our patient however was the presence of VITT.

Another case report of 33-year-old patient with past medical history of obesity and dyslipidaemia presented with inferior ST elevation and reciprocal changes in leads 1 and AVL. CAG revealed 83% stenosis with a heavy thrombus burden in the middle segment of the left circumflex artery and TIMI 2 flow was obtained after repeated attempts at aspiration and administration of tirofiban. He was found to have thrombocytopaenia and raised D-dimer and was diagnosed with VITT and passed away during hospital admission. The patient in our case did not have any angiographically visible coronary stenosis [8].

It is therefore important to consider the diagnosis of VITT in patients presenting with venous and arterial thrombosis in setting of COVID-19 infection. The National Institute for Health and Care Excellence (NICE) in the United Kingdom recommends commencing intravenous immunoglobulin (IVIG) and the International Society on Thrombosis and Haemostasis (ISTH) recommends administering steroids if a patient’s platelet count is less than 50 × 10^9^/l. Both guidelines recommend strict monitoring of fibrinogen levels and should be kept >1.5 g/l. Plasma exchange can be considered in patients with platelet count < 30 × 10^9^/l despite IVIG and steroids [8,19].

## Conclusions

In conclusion, vaccine-induced thrombocytopaenia and thrombosis is an uncommon condition however has been reported. COVID-19 vaccines are associated with both venous and arterial thrombosis. Our patient had acute myocardial infarction due to heavy thrombus burden and his course of disease was complicated by development of vaccine induced thrombocytopaenia and thrombosis syndrome. The outcome usually is unfavourable in these patients and our patient also did not survive unfortunately. It is important for clinicians to be aware of this condition, in particular in patients presenting with acute myocardial infarction within few days of receiving COVID-19 vaccination.
